# A Typology of Existing Machine Learning–Based Predictive Analytic Tools Focused on Reducing Costs and Improving Quality in Health Care: Systematic Search and Content Analysis

**DOI:** 10.2196/26391

**Published:** 2021-06-22

**Authors:** Ariadne A Nichol, Jason N Batten, Meghan C Halley, Julia K Axelrod, Pamela L Sankar, Mildred K Cho

**Affiliations:** 1 Stanford School of Medicine Stanford Center for Biomedical Ethics Stanford, CA United States; 2 Department of Medical Ethics and Health Policy Perelman School of Medicine Philadelphia, PA United States

**Keywords:** machine learning, artificial intelligence, ethics, regulation, health care quality, costs

## Abstract

**Background:**

Considerable effort has been devoted to the development of artificial intelligence, including machine learning–based predictive analytics (MLPA) for use in health care settings. The growth of MLPA could be fueled by payment reforms that hold health care organizations responsible for providing high-quality, cost-effective care. Policy analysts, ethicists, and computer scientists have identified unique ethical and regulatory challenges from the use of MLPA in health care. However, little is known about the types of MLPA health care products available on the market today or their stated goals.

**Objective:**

This study aims to better characterize available MLPA health care products, identifying and characterizing claims about products recently or currently in use in US health care settings that are marketed as tools to improve health care efficiency by improving quality of care while reducing costs.

**Methods:**

We conducted systematic database searches of relevant business news and academic research to identify MLPA products for health care efficiency meeting our inclusion and exclusion criteria. We used content analysis to generate MLPA product categories and characterize the organizations marketing the products.

**Results:**

We identified 106 products and characterized them based on publicly available information in terms of the types of predictions made and the size, type, and clinical training of the leadership of the companies marketing them. We identified 5 categories of predictions made by MLPA products based on publicly available product marketing materials: disease onset and progression, treatment, cost and utilization, admissions and readmissions, and decompensation and adverse events.

**Conclusions:**

Our findings provide a foundational reference to inform the analysis of specific ethical and regulatory challenges arising from the use of MLPA to improve health care efficiency.

## Introduction

### Background

Machine learning–based predictive analytics (MLPA) products are emerging as a strategy for controlling rising health care costs [1]. Advanced statistical analyses have long been used to estimate the likelihood of future health outcomes based on previous events and inform clinical and administrative decisions. MLPA offers a benefit over current approaches because of its ability to draw from larger and more diverse electronic health record (EHR) data sets and potentially draw inferences without human involvement in defining input variables to predict outcomes, with the goal of improving speed and accuracy [2,3]. A particular aspect of health care seen as especially ripe for MLPA application is health care efficiency, which improves patient outcomes while reducing health care costs [4]. By gaining insights from large amounts of clinical information stored in EHR systems, MLPA is being used to identify and direct resources toward patients at higher risk of poor outcomes.

Incentives for health systems to adopt products focused on health care efficiency stem—at least in part—from federal policies and payment structures that encourage value-based care under the Affordable Care Act and value-based purchasing and bundling programs instituted by the Centers for Medicare & Medicaid Services (CMS). For example, the CMS Hospital Readmissions Reduction Program reduces reimbursements to hospitals with excess unplanned 30-day hospital readmissions for certain health conditions [5]. Linking CMS payments to the quality of care creates a financial incentive for health systems to adopt MLPA products that aim to improve health care efficiency.

However, experts recognize that although MLPA could improve the efficiency of delivered care, its use in the health care domain poses distinct ethical challenges because of its lack of transparency, continuous adaptation without human intervention, and its potential for systematic error leading to unfair decisions or actions [6-8]. These challenges have been demonstrated by high-profile cases, such as the predictive risk-stratification algorithm developed by Optum, which resulted in significant racial bias against Black patients when health costs were used as a proxy measure of health needs [9]. Obtaining, sharing, and handling the sensitive data necessary for MLPA in health care also raises privacy concerns. In another high-profile case, technology giant Google was sued for alleged violation of the Health Insurance Portability and Accountability Act in its handling of patient records from the University of Chicago Medical Center for the development of predictive artificial intelligence tools [10]. More recently, Google’s partnership with Ascension, one of the largest private, faith-based health care systems in the United States, came under investigation after revealing that the tech company had obtained protected health information without patient consent [11,12]. Many of the ethical challenges mentioned, such as privacy concerns, are not unique to the utilization of MLPA and are relevant to other uses of advanced statistical analyses implemented in health care. However, continuous self-learning and the lack of transparency in MLPA algorithms are two unique aspects of the techniques that make it difficult to evaluate the models and understand how decisions are made.

These cases also highlight the importance and challenges of oversight of these complex software products. Unlike drugs and medical devices that the Food and Drug Administration (FDA) typically regulates, MLPA-based products are constantly and inherently mutable, complicating the definition of the final product. The US FDA is actively testing a regulatory framework for software as a medical device through a precertification pilot program. The framework shifts the emphasis away from the evaluation of completed products to the evaluation of processes that demonstrate a “culture of quality and organizational excellence” [13,14]. It is unclear whether or how such a framework applies to MLPA products to improve health care efficiency or what features constitute a *culture of quality and organizational excellence* capable of facilitating the development of safe and effective products. This ambiguity is because of, at least in part, a lack of systematic information on the characteristics of organizations that develop such MLPA, which is essential to understanding their potential ability to self-regulate. The diversity of expertise required to develop MLPA for health care, including computer science, software engineering, and medicine, suggests that teams brought together to develop and implement particular products will include members who are unfamiliar with the norms and culture of biomedical research and development and clinical practice [15,16]. The types of organizations developing MLPA products and the types of expertise at the organizations are largely unknown, precluding analysis of the alignment of interests and expertise with the needs of patients and health care providers [17].

### Objective

The main objective of this study is to map the landscape of currently available MLPA products marketed with the aim of improving health care efficiency. The study also seeks to characterize organizations developing these MLPA products, with the subsequent goal of identifying relevant ethical, regulatory, and policy implications.

## Methods

### Search Strategy

We sought to identify MLPA products based on publicly available marketing information. To identify these products, we assessed 4 databases: LexisNexis, PubMed, Web of Knowledge, and Indeed.com. PubMed references frequently omitted necessary details to judge a product’s current use, and many of the results were duplicative with Web of Knowledge results. On this basis, we eliminated PubMed and conducted our research using the other 3 databases. LexisNexis searches returned the highest number of nonduplicative results. Indeed.com (the world’s largest job listing website) and Web of Knowledge were used because they returned additional nonduplicative results. Search terms such as “hospitals,” “health care organizations,” “machine learning,” and “predictive analytics” were used (see Multimedia Appendix 1 for further details). Search terms were tested, reviewed, and refined to maximize the number of returned MLPA products that met our inclusion criteria. LexisNexis search parameters included the United States and English language, and a date range from April 1, 2015, to February 1, 2019, selected to capture efforts launched likely in response to April 2015 American Recovery and Reinvestment Act-mandated changes to CMS reimbursement policies.

### Eligibility Criteria and Screening Process

We first removed all duplicates and any results that did not mention specific products (ie, congressional transcripts). For the identified products, we conducted additional targeted searches as needed to elucidate whether specific products met the eligibility criteria. The final list of eligible products for which the marketing materials were identified made the following claims: (1) the MLPA product made health care–related predictions, (2) the product primarily aimed to improve health care quality and reduce costs (ie, improve health care efficiency), (3) the product used EHR-sourced data, and (4) the product had been implemented by an identifiable US health system or provider, and possibly, though not necessarily, utilized on a routine basis. In addition, we excluded products if, based on marketing language, they (1) lacked a predictive element, (2) were not directly related to improving the quality of delivered care (eg, managing appointment schedules), or (3) solely used patient data that were not EHR-sourced (eg, data from a wearable device).

### Data Extraction

For all remaining products, we used the product website to collect additional information about the product characteristics and the organization that developed it. Characteristics included health care partners using the product, sources of data used to create and train the MLPA algorithms, and the type and size of the organization marketing the product. We also characterized the companies by the number of employees, the type of business, and whether the chief executives or board members had a clinical degree, including doctor of medicine, registered nurse, or other.

### Data Analysis

We used content analysis to generate MLPA product categories based on the type of prediction made [18]. To do this, we first generated initial codes from the verbatim marketing language available on the organization websites. If a single product made multiple predictions, we applied multiple codes to capture all the product predictions, and 2 researchers independently coded 50% of the products. All discrepancies were resolved through discussion with the full research team. A single researcher coded the remaining products. After all MLPA products received initial codes, we grouped the initial codes into overarching categories based on the prediction type. We counted the total number of products in each category as well as membership in multiple categories.

## Results

### Search Results and Characterization of Companies

**Figure 1:**
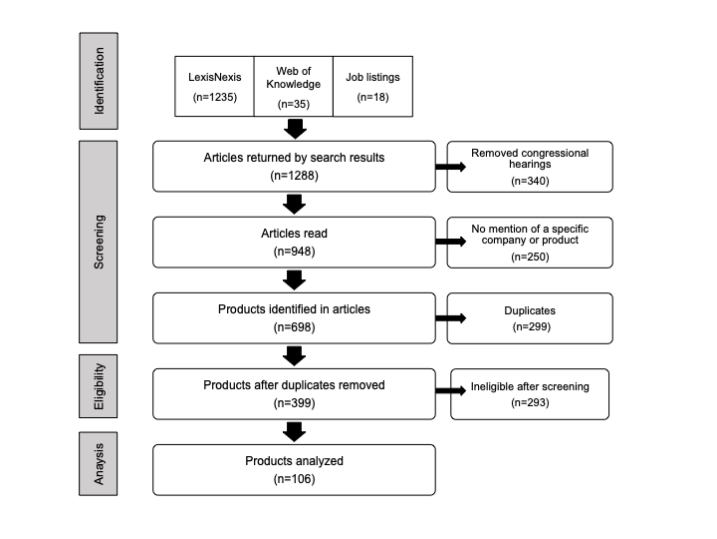
Identification of machine learning–based predictive analytics products.

From 1288 articles and other sources, we found 106 MLPA products developed by 96 companies that met our inclusion and exclusion criteria (Figure 1). The products and characteristics of the companies are listed in Table 1.

**Table 1:** Characteristics of companies developing machine learning–based predictive analytics products (N=96).

Characteristics and categories		Values, n (%)
**Organization size**		
	Small (1-50 employees)	34 (35)
	Medium (51-1000 employees)	25 (26)
	Large (more than 1000 employees)	37 (39)
**Organization type**		
	Computer software company—health care	68 (71)
	Computer software company—general	14 (15)
	Health insurer	6 (6)
	Provider (hospital or health system)	8 (8)
**CEO^a^ with a clinical degree**		
	Yes	15 (16)
	No	81 (84)
**C-suite or board member with a clinical degree**		
	Yes	62 (65)
	No	34 (35)

^a^CEO: chief executive officer.

Many organizations did not meet the inclusion criteria because their products were not yet implemented by an identifiable health system or provider. Other organizations were similarly ineligible because the marketing language did not claim that their product used MLPA for predicting how to reduce cost and improve quality of care. Of the organizations, 92% (88/96) developed 1 product that met the inclusion criteria, whereas 8% (8/96) had more than one product. Companies were broadly distributed in terms of size. The vast majority 85% (82/96) were computer software companies, of which 83% (68/82) specialized in health care–related products. Of the MLPA developers, 15% (14/96) were health insurers, hospitals, or health systems.

Although chief executive officers (CEOs) of 84% (81/96) of companies did not have a clinical degree, 65% (62/96) listed a C-suite or board member who did. Of the software companies specializing in health care, 16% (11/68) had a clinician CEO, and 72% (49/68) had a clinician C-suite or board member. Computer software companies specializing in health care made up 94% (32/34) of small organizations with 50 employees or less. None of the large general computer software companies had a clinician as CEO, 75% (9/12) had a chief medical officer, and 8% (1/12) had a clinician C-suite or board member. All providers (hospitals or health systems) were large organizations with more than 1000 employees. Of the providers, 50% (4/8) had a clinician as CEO, and all providers had a clinician C-suite or board member.

### Classification of MLPA Products

#### Overview

We identified 5 categories of predictions made by MLPA products based on the publicly available product marketing materials: disease onset and progression, treatment, cost and utilization, admissions and readmissions, and decompensation and adverse events (Table 2).

Of the products, 67% (71/106) were assigned to more than one category. A full list of products and their assigned categories can be found in Multimedia Appendix 2. Here, we describe the categories qualitatively and describe a typical product from each category.

**Table 2:** Categories of predictions made by MLPA products.

MLPA^a^ prediction category^b^	Examples of specific predictions	Example quotes from product descriptions provided by developers
Disease onset and progression predictions (n=62)	Patient outcome; unspecified diseases; chronic illnesses; specified diseases; mortality; comorbidities	“Enables early prediction of disease onset.” “Clinicians can now see red flags for admitted patients at elevated risk of mortality three to five days in advance.”
Treatment predictions (n=48)	Best course of treatment; candidates for palliative care or hospice; untreated or undertreated individuals (often referred to as *gaps in care*); expected recovery trajectory; type of care required; best medication or drug efficacy; patients at risk of receiving unnecessary clinical care (visits, tests, procedures, or antibiotics); next steps of medical care that a physician would order	“Identify members earlier in their disease progression who are likely going to be overmedicalized during the last 6-12 months of life.” “Helps clinicians make data-driven decisions about a patient’s care plan.”
Cost and utilization predictions (n=38)	High-cost members of a population; high utilizers in a population; risk stratification; cost of caring for a specific patient; Medicare’s predicted risk	“Predict health care cost for individuals for customer specified time periods.”
Decompensation and adverse events predictions (n=34)	Hypotensive event; sepsis; hemodynamic instability; inpatient or outpatient decompensation; postoperative complications or surgical site infections; risk of adverse event; adverse medication reactions; hospital-acquired infection; hospital-acquired pressure injury	“Identify patients at risk of surgical site infection.” “A respiratory failure detection algorithm...can highlight patients at a higher risk of prolonged ventilation up to 48 hours before onset.”
Admissions and readmissions predictions (n=33)	Readmission risk; avoidable hospital admission or readmission or ED^c^ use; unplanned ICU^d^ admission or readmission; ED presentation volume; hospitalization; patient flow; length of stay or risk of an extended length of stay; discharge date; disposition at the end of hospitalization	“Predicted output is the % chance that the patient will not return/be readmitted.” “Using only six vital signs and patient age, our machine learning tool more accurately predicted down-transfer success.”

^a^MLPA: machine learning–based predictive analytics.

^b^Totals do not add up to 106 because categories are not mutually exclusive.

^c^ED: emergency department.

^d^ICU: intensive care unit.

#### Disease Onset and Progression

A total of 62 products were used to predict the disease onset and progression (see Textbox 1 for an example product). The marketing language for some of these products did not specify particular conditions or diseases. For products that did specify diseases or health states, 22 products were identified as predicting the onset and development of diabetes, cancer, or cardiovascular conditions. In addition, 5 of those 22 products predicted more than one of the 3 listed conditions. Nearly half of these products also explicitly performed cost and utilization prediction instead of simply providing data that could be used to reduce cost.

**Example of a disease onset and progression product.**

**Medictiv by CitiusTech**

- CitiusTech is a large private health care information technology company. Medictiv is a statistical analysis tool advertised as having machine learning capabilities to analyze longitudinal electronic health record–sourced data to predict the onset and progression of various unspecified diseases. Medictiv also advertises specific use cases for chronic kidney disease (CKD) and diabetes. For CKD, Medictiv uses longitudinal patient and laboratory data to predict disease progression risk for CKD stage 3 patients. For diabetes, Medictiv uses data available within 72 hours of admission, including laboratory results, demographic data, comorbidities, and health insurance claims to predict patients’ length of stay, risk of readmission, and risk stratification [19].

#### Treatment

A total of 48 products made predictions related to patient treatment (see Textbox 2 for an example product). The most common type of prediction was identifying patients with *care gaps* or who were *untreated* or *undertreated*. The available marketing language does not specify the meaning of these terms. Nearly all products identifying care gaps explicitly mentioned performing a cost and utilization prediction. In the treatment category, other products made predictions meant to aid clinicians in therapeutic decision making (eg, identifying the best medication to use, predicting adverse reactions, predicting drug interactions, and predicting unnecessary antibiotic use). A large proportion (29/48, 60%) of the products in the treatment category were also categorized as predicting disease onset and progression.

**Example of a treatment product.**

**Identifi by Evolent Health**

- Identifi is Evolent Health’s value-based care product, which aims to reduce costs and improve the quality of delivered care. Identifi’s machine learning–based predictive analytics algorithms use clinical, social, and administrative data to predict the best course of treatment for a patient and identify gaps in a patient’s care. They also make predictions about patient outcomes, risk of readmission, and risk stratification. Evolent Health is a public health care company with between 1000 and 5000 employees. It advertises Identifi to providers and health plans [20].

#### Cost and Utilization

This category comprises products whose MLPA algorithms predict the cost or utilization of health care (n=38; see Textbox 3 for an example product). As one of our inclusion criteria was a focus on improving health care efficiency, all products in our sample had the goal of reducing costs. However, only products that aimed to reduce costs by making explicit predictions about costs and utilization were included in this category. As there were numerous products that used MLPA primarily to predict admissions or readmissions (n=33), they were assigned their own category (described below and not included in the n=38 of the cost and utilization category). In the cost and utilization category, financial risk stratification of patient populations was the most common MLPA use, with a wide margin. Other common use cases were predicting which patients would be high cost or become high utilizers and predicting Medicare’s predicted risk. Half of the products in this category also fell into the categories of disease onset, progression predictions, and treatment predictions.

**Example of a cost and utilization product.**

**Waystar Platform by Waystar**

- Waystar uses social determinants of health, along with hospital and consumer data, to stratify the patient population according to risk and cost [21]. The company also helps with revenue integrity by identifying incorrectly coded and undercoded claims to help providers maximize revenue. Waystar is a medium-sized private information technology company with 500-1000 employees.

#### Decompensation and Adverse Events

The products in this category (n=34) were designed to act as early warning systems for the occurrence of adverse events or decompensations (see Textbox 4 for an example product). We grouped decompensation and adverse event predictions together when we defined our prediction categories because of the frequent overlap in the clinical application of the products. Algorithms typically use vital signs combined with EHR data to closely monitor in-patient populations and alert care teams for decompensation. Products alerting for general inpatient decompensation were the most common in this category (n=18), followed by early warning systems for sepsis (n=14). Monitoring for an outpatient decline, hospital-acquired infections, and postoperative complications were also quite common. The decompensation and adverse events category had the least overlap with other categories.

**Example of a decompensation and adverse events product.**

**InSight by Dascena**

- Dascena’s InSight is a paradigmatic application of machine learning–based predictive analytics used to provide an early warning of an adverse event. Dascena is a small, private company with less than 50 employees. The InSight algorithm warns of sepsis onset using vital sign data located in patients’ electronic health records, which is typical of products in this category. InSight provides physicians with real-time alerts and boasts its ability to forecast a patient’s condition 4 hours in the future [22].

#### Admissions and Readmissions

In this category (n=33), predicting the risk of readmission was the most common application (n=21), where the marketing language had to explicitly state *predicting risk of readmission* as a use case of the product (see Textbox 5 for an example product). Other admissions predicted length of stay and intensive care unit occupancy. More than half of the products predicting admissions and readmissions fell into the disease onset and progression category; overlap with the treatment category was also fairly high (8/33, 24%).

**Example of an admissions and readmissions product.**

**Conduent’s Midas Readmission Penalty Forecaster**

- Midas Readmission Penalty Forecaster is a common product developed in response to Centers for Medicare & Medicaid Services Hospital Readmissions Reduction Program [23]. Conduent’s white pages market the product as a web-based tool to forecast 30-day unplanned readmissions to help health care organizations predict penalties and adjust their care delivery. Midas Readmission Penalty Forecaster estimates total readmissions, excess readmissions, and financial penalties for 6 Centers for Medicare & Medicaid Services cohorts: patients diagnosed with acute myocardial infarction, heart failure, pneumonia, or chronic obstructive pulmonary disease or patients receiving a coronary artery bypass graft, total hip arthroplasty, or total knee arthroplasty. This product was developed by Conduent, an information technology company with more than 10,000 employees working in health care and 20 other industries, ranging from insurance and government to casinos, oil, and gas.

## Discussion

### Principal Findings

Our results provide an overview of the emerging MLPA applied to improve health care efficiency and provide a systematic categorization of actual applications of this technology in patient care. The products identified as being currently in use are predominantly marketed by computer software companies. Our results also provide a systematic framework for mapping the characteristics of organizations operating in the field of MLPA in health care and the products they produce, based on the specific predictions that these products are intended to provide in a health care setting.

The potential for MLPA to transform health care has generated much anticipation to the possibilities for this technology to improve health care quality and reduce costs. Bates et al [2] previously forecasted 6 likely targets of predictive analytics in health care: high-cost patients, readmissions, decompensation, adverse events, triage, and diseases affecting multiple organ systems. Our results suggest that many of these uses materialized in the markets. For example, our results confirmed a large market presence of MLPA products that aim to predict hospital readmission within 30 days of initial discharge as well as decompensation and adverse events. In addition, among the MLPA products categorized as predicting disease onset and progression within our framework, diabetes, cancer, or cardiovascular conditions were the most common—conditions all affecting multiple organ systems. Furthermore, our categories focused on prediction of cost and utilization as well as on prediction of treatment; both have close ties to the previously forecasted focus of predictive analytics on high-cost patients and triage in health care. However, our framework also goes beyond these original predictions by providing a systematic, evidence-based approach to mapping the field of MLPA products in health care organized around the specific predictions provided by these products instead of the intended use or target population.

Our results also suggest that MLPA products are increasingly being used in response to CMS reimbursement policies. The readmissions predictions may reflect a response to the recent CMS Hospital Readmissions Reduction Program, which reduces payments to hospitals with excessive numbers of readmissions [5]. The focus on diabetes, cancer, or cardiovascular conditions in MLPA products identified in our analysis maps directly to conditions subjected to bundled payments under the CMS’s Bundled Payments for Care Improvement initiative [24]. In addition, products marketed as predicting high-cost patients (while also identifying some additional applications, such as predicting Medicare’s predicted risk score) likely emerged, at least in part, in response to Medicare’s reimbursement policies transitioning from fee-for-service to risk-adjusted fixed payments per episode of care [25]. Although the use of MLPA to respond to shifting reimbursement policies is perhaps unsurprising, it also raises questions about the alignment of these financial incentives with the goal of improving patient care. These goals address critically important needs of the health care system in the United States, but trying to meet them can raise ethical issues. Improving care quality and outcomes without increasing costs poses a myriad of challenges. Thus, when efficiency is improved by reducing costs, there are concerns that quality of care has been negatively affected. Although the aims of improving quality and cost are ideally aligned with the stated goals of these MLPA products, it is difficult to know whether this is indeed the case when employing MLPA products without further information on how the underlying models are developed and implemented in the clinical setting. Moreover, many MLPA algorithms have not been rigorously tested, and little is known about their comparison with other predictive analytics or clinical judgment. Evaluation of MLPA algorithms is particularly difficult given the opacity of the models and their *black box* nature.

Our results also provide an essential framework for considering various approaches to regulation in this diverse and rapidly changing marketplace. The FDA is currently developing a framework that incorporates the level of risk to the patient in its review process. Having a systematic framework of categories that may reflect varying degrees or types of risk to patients (eg, treatment recommendations vs prediction of health care costs) may therefore be important. Traditionally, software products have not been subjected to the level of regulatory scrutiny applied to drugs or medical devices, nor has the technology sector established processes for identifying or evaluating ethical issues that may arise from their products. Developing an effective regulatory framework requires an understanding of various stakeholders and organizations involved in this marketplace, potential sources of conflict, and the resources necessary for success. In examining MLPA products, which inherently change and adapt as they incorporate new data, regulators may need to consider the extent to which business requirements—including production schedules, fundraising, and profit goals—are aligned with the design process.

In addition, further examination is needed regarding the role of clinical expertise within these companies in light of the FDA’s self-regulation approach in evaluating companies based on *a culture of quality and organizational excellence*. There is a relative dearth of clinical training among CEOs and others in company leadership. Of the organizations we identified, only 1 in 6 was led by a clinical degree-holding CEO, and more than a third did not have a clinician in the C-suite or on their board of trustees. Although clinicians may be involved in different roles, they are underrepresented in the highest leadership positions, which may have implications for the level of awareness that a company has of the norms and culture of biomedical research and clinical practice. The influence of business requirements and expertise may also vary depending on the size of the company: although small health care technology startups might be under more significant financial and time pressures with the need to raise venture capital, larger companies likely have more resources to draw from. However, our analysis suggests that large companies are also less likely to specialize in health care software or technology and less likely to have a clinician in a leadership position than small health care technology startups. More research is needed to determine the extent to which factors such as company size, business requirements, and clinical expertise influence the design and implementation of MLPA in health care and their potential importance in designing regulatory frameworks.

Our study has several limitations. Our results are limited by our reliance on publicly available web-based information, such as product websites, press releases, and health system websites. Products developed by nonprofit health systems, academic institutions, or large insurers may not have been readily identifiable, as their products are often not marketed externally. Therefore, we are less likely to have identified products developed by a health system or health insurer that are not sold for use in other systems. Another limitation is that the predictions were categorized based on the marketing language used by the companies to describe their own products, so the actual extent to which these products do what they are marketed to do remains unclear. In addition, we do not know how often the tools are used by the health care system where they are implemented. Some may be used frequently and others rarely.

### Conclusions

There is a rapidly emerging set of products that utilize MLPA with the dual goals of improving health care and addressing cost containment. These goals address critically important needs of the health care system in the United States. Improving care quality and outcomes is not necessarily at odds with lowering costs. There is an underlying ethical tension, however, when health care efficiency is improved by reducing cost with possible negative effects on quality. How MLPA developers perceive these trade-offs and whether reliance on such tools may exacerbate discrimination based on underlying biases is difficult to assess using currently available data. The significant role of the software and technology companies, which might have little experience in understanding clinical care, using health data, or applying medical ethics or law, suggests that regulatory approaches that rely on self-regulation and organizational culture may be challenging for the evaluation of MLPA products. More research on the process of developing these novel tools is needed to further assess the implications for policy and regulation.

## Appendix

Multimedia Appendix 1 Search terms.

Multimedia Appendix 2 Categorization of machine learning–based predictive analytics products.

## Abbreviations

CEO: chief executive officer
CMS: Centers for Medicare & Medicaid Services
EHR: electronic health record
FDA: Food and Drug Administration
MLPA: machine learning–based predictive analytics
