# Supplementary material for: A Typology of Existing Machine Learning–Based Predictive Analytic Tools Focused on Reducing Costs and Improving Quality in Health Care: Systematic Search and Content Analysis
Source: J Med Internet Res. 2021 Jun 22;23(6):e26391. doi: 10.2196/26391 (PMC8277386; doi:10.2196/26391)
Supplement: Multimedia Appendix 1 [file jmir_v23i6e26391_app1.doc]

**Appendix 1: Search Terms**

A second, supplementy search of Web of Knowledge was conducted to provide coverage of scientific and academic activity. Web of Knowledge search parameters were: 2016-2018, United States, and articles. The “refine” function on Web of Knowledge was used to generate a list of organizations associated with the articles and documents returned by the Web of Knowledge search.

A third, supplementary search of full-time job listings was conducted on Indeed.com on 8/17/18 to capture the latest organizations hiring in this field. Results were reviewed by organization. On the basis of the number of relevant jobs listed, the top 15 organizations were selected. A few additional products were discovered while extracting data from the above searches and these products were added to the data set.

| Database | Search Terms |
| --- | --- |
| LexisNexis | “predictive analytics” AND “patient” AND “health care or health or hospital” |
| Web of Knowledge | “hospitals OR health care organizations OR health care organizations” with either AND “machine learning” or AND “predictive analytics” |
| Indeed.com | “machine learning” and “health care” |
