# Supplementary material for: A Typology of Existing Machine Learning–Based Predictive Analytic Tools Focused on Reducing Costs and Improving Quality in Health Care: Systematic Search and Content Analysis
Source: J Med Internet Res. 2021 Jun 22;23(6):e26391. doi: 10.2196/26391 (PMC8277386; doi:10.2196/26391)
Supplement: Multimedia Appendix 2 [file jmir_v23i6e26391_app2.doc]

**Appendix 2: Categorization of MLPA Products**

| **Company** | **Product** | **Disease Onset and Progression Predictions** | **Treatment Predictions** | **Cost and Utilization Predictions** | **Decompensation and Adverse Events Predictions** | **Admissions and Readmissions Predictions** |
| --- | --- | --- | --- | --- | --- | --- |
| Buoy (in collaboration with CVS Health) | Buoy | ✓ | ✓ |  |  |  |
| AlertWatch | AlertWatch OB |  |  |  | ✓ |  |
| AlertWatch | AlertWatch OR |  |  |  | ✓ |  |
| AMP3D | CoMET (Continuous Monitoring of Event Trajectories) | ✓ |  |  | ✓ | ✓ |
| Astarte Medical Partners | NICUtrition MAGI | ✓ |  |  |  |  |
| BaseHealth (in collaboration with Banner Health) | BaseHealth Predictive RAF Analytics |  |  | ✓ | ✓ |  |
| Cardinal Analytx Solutions | Cardinal Analytx Cost Bloom |  |  | ✓ |  |  |
| Care at Hand | Care at Hand proprietary algorithm |  |  | ✓ |  | ✓ |
| Clarify Health Solutions | Clarify Care Prism | ✓ |  | ✓ |  |  |
| Cloud Medx | Cloud Medx | ✓ | ✓ | ✓ |  |  |
| Dascena | AutoTriage |  |  |  | ✓ | ✓ |
| Dascena | Previse | ✓ |  |  |  |  |
| Dascena | InSight |  |  |  | ✓ |  |
| Excel Medical | Visensia WAVE platform | ✓ |  |  | ✓ |  |
| Fifth Eye | Analytic for Hemodynamic Instability |  |  |  | ✓ |  |
| H2O.ai | H2O.ai |  | ✓ |  | ✓ | ✓ |
| HarrisLogic | StellaCare |  | ✓ |  |  | ✓ |
| HBI Solutions | HBI Spotlight | ✓ | ✓ |  |  |  |
| Kryptowire | Warfighter Analytics platform | ✓ |  |  | ✓ |  |
| Livongo Health | Livongo | ✓ | ✓ | ✓ |  |  |
| Lumiata | Health Data Platform | ✓ |  | ✓ |  |  |
| Medalogix | Medalogix Bridge | ✓ | ✓ |  |  |  |
| Medalogix | Medalogix Nuture |  |  |  | ✓ | ✓ |
| MYnd Analytics, Inc. | MYnd Analytics PEER Report |  | ✓ |  |  |  |
| NFANT Labs | NFANT Analytics | ✓ |  |  |  |  |
| Parallax Health Sciences, Inc. | Acuity | ✓ |  | ✓ |  |  |
| PeraHealth | Rothman Index Analytics | ✓ | ✓ |  | ✓ | ✓ |
| PeraHealth | Rothman Index TREND |  |  |  | ✓ |  |
| Quire | Quire | ✓ |  |  |  |  |
| Qventus | Qventus |  |  |  |  | ✓ |
| Raiven Healthcare | Raiven platform | ✓ | ✓ | ✓ |  |  |
| Remedy | Sentinel | ✓ |  | ✓ | ✓ |  |
| Senscio Systems | Ibis | ✓ |  |  | ✓ | ✓ |
| Tridiuum | Tridiuum1 |  | ✓ |  |  |  |
| Turn-Key Health | Palliative Illness Management |  | ✓ | ✓ |  |  |
| VigiLanz | VigiLanz Temporalytics | ✓ | ✓ |  | ✓ |  |
| VitreosHealth | VitreosHealth | ✓ |  | ✓ |  | ✓ |
| Vivify Health | Vivify Health | ✓ | ✓ |  | ✓ |  |
| Wanda, Inc. | Wanda Health Management Platform | ✓ |  |  | ✓ |  |
| Jvion | Jvion Cognitive Machine | ✓ | ✓ |  | ✓ | ✓ |
| Alignment Healthcare | Alignment Health Plan | ✓ |  |  | ✓ |  |
| Apixio | Iris | ✓ |  | ✓ |  |  |
| Arcadia.io | Arcadia Analytics platform |  | ✓ | ✓ |  |  |
| axialHealthcare | axialINSIGHT | ✓ |  |  |  |  |
| AxisPoint Health | CarePoint | ✓ | ✓ |  |  | ✓ |
| Ayasdi | Ayasdi Care |  | ✓ | ✓ |  |  |
| Castlight Health | Engage |  | ✓ | ✓ |  |  |
| Catasys, Inc. | OnTrak solution |  | ✓ | ✓ |  |  |
| Clover Health | Clover Health | ✓ | ✓ |  |  |  |
| DataRobot | DataRobot |  |  |  |  | ✓ |
| IBC | Informatics | ✓ |  |  |  | ✓ |
| Koan Health | Datalyst |  | ✓ | ✓ |  |  |
| Lumeris (acquired Forecast Health) | Collaborative Payer Provider Model | ✓ | ✓ | ✓ | ✓ |  |
| MedeAnalytics, Inc. | MedeAnalytics Population Health Platform | ✓ | ✓ | ✓ |  |  |
| Medopad | Medopad | ✓ |  |  |  |  |
| Palantir Health | Palantir Health | ✓ | ✓ |  |  |  |
| Pieces Technologies | Pieces Decision Sciences |  |  |  | ✓ | ✓ |
| Prognos | Prognos | ✓ | ✓ | ✓ |  |  |
| Quantzig | Quantzig Big Data Analytics | ✓ | ✓ |  |  | ✓ |
| Teletracking | Teletracking |  |  |  |  | ✓ |
| The Garage | ROOT | ✓ |  |  |  |  |
| VirtualHealth | Helios |  | ✓ | ✓ |  |  |
| Waystar | Waystar | ✓ |  | ✓ |  |  |
| welldoc | BlueStar | ✓ |  |  |  |  |
| HCA Healthcare (in collaboration with Digital Reasoning) | Artificial Intelligence- Powered Oncology Software | ✓ | ✓ | ✓ |  |  |
| Aetna (in collaboration with Merck) | AetnaCare | ✓ | ✓ |  |  |  |
| Aetna (in collaboration with Apple) | Attain |  | ✓ |  |  |  |
| Allscripts | Avenel |  |  | ✓ |  |  |
| Cerner | Cerner Population Health Management Platform |  | ✓ |  | ✓ | ✓ |
| Cigna | Cigna | ✓ |  |  |  |  |
| CitiusTech | Medictiv | ✓ |  | ✓ |  | ✓ |
| Cognizant | TriZetto TranZform Analytics |  | ✓ | ✓ |  |  |
| Conduent Business Services | Conduent Midas Readmission Penalty Forecaster |  |  |  |  | ✓ |
| Cotiviti | DxCG Intelligence | ✓ | ✓ | ✓ |  |  |
| CVS HEALTH (in collaboration with IBM Watson Health) | CVS HEALTH Caremark Pharmacy Benefits Management | ✓ |  | ✓ |  |  |
| Dell Services | Dell EMC |  |  |  | ✓ |  |
| Edwards Lifesciences | HemoSphere |  |  |  | ✓ |  |
| Edwards Lifesciences/Bay Labs | CardioCare | ✓ |  |  |  |  |
| Epic | Epic | ✓ |  |  | ✓ | ✓ |
| Evolent Health | IdentifiSM | ✓ | ✓ | ✓ |  | ✓ |
| EXL | CareRadius | ✓ | ✓ | ✓ |  |  |
| GE Healthcare | Command Center Wall of Analytics |  |  |  |  | ✓ |
| Healogics | Healogics |  | ✓ | ✓ |  |  |
| Health Catalyst | Health Catalyst Analytics Platform | ✓ | ✓ |  | ✓ | ✓ |
| IBM | Watson Care Manager | ✓ | ✓ |  |  |  |
| Inovalon | Inovalon’s Natural Language Processing as a Service (NLPaaS) |  |  | ✓ |  |  |
| Insight | Insight |  | ✓ |  |  |  |
| IOMICS Intelligent Analytics | FUSION | ✓ | ✓ |  |  |  |
| IQVIA | IQVIA CORE | ✓ | ✓ |  |  |  |
| Johns Hopkins HealthCare Solutions | ACG System | ✓ |  | ✓ |  | ✓ |
| Microsoft Azure | Risk-O-meter/Readmission score as service | ✓ |  |  |  | ✓ |
| Montefiore Health System | Montefiore Einstein AI platform | ✓ |  |  |  |  |
| Mount Sinai Health System | Mount Sinai Health System Platform | ✓ |  |  | ✓ |  |
| NorthShore University HealthSystem | Clinical Analytics Predictive Engine | ✓ |  |  |  | ✓ |
| NTT Data | Business Intelligence & Analytics for Health Plans |  |  | ✓ | ✓ | ✓ |
| Oracle | APOLLO | ✓ | ✓ | ✓ |  |  |
| Penn Medicine Predictive Healthcare | Heart Failure | ✓ |  |  |  | ✓ |
| Penn Medicine Predictive Healthcare | Palliative Connect |  | ✓ |  |  |  |
| Penn Medicine Predictive Healthcare | Sever Sepsis / Septic Shock Early Warning System 2.0 |  |  |  | ✓ |  |
| Philips Research | eICU (IntelliSpace Console hosted on HealthSuite) |  |  |  | ✓ |  |
| Philips Research | IntelliVue |  |  |  | ✓ |  |
| SAS Industry Solutions | SAS AI solutions | ✓ | ✓ | ✓ |  | ✓ |
| SCIOInspire, Corp. | SCIOVantage |  | ✓ |  |  | ✓ |
| TIBCO Software, Inc. | TIBCO Data Science |  |  |  | ✓ |  |
| United Healthcare Group | Optum | ✓ | ✓ | ✓ |  | ✓ |
| UnityPoint Health | UnityPoint Health |  | ✓ |  | ✓ | ✓ |
